# Sibling sRNA RyfA1 Influences *Shigella dysenteriae* Pathogenesis

**DOI:** 10.3390/genes8020050

**Published:** 2017-01-26

**Authors:** Megan E. Fris, William H. Broach, Sarah E. Klim, Peter W. Coschigano, Ronan K. Carroll, Clayton C. Caswell, Erin R. Murphy

**Affiliations:** 1 Department of Biological Sciences, Ohio University, 1 Ohio University Drive Athens, Athens, OH 45701, USA; mf590113@ohio.edu (M.E.F.); sk231407@ohio.edu (S.E.K.); carrolr3@ohio.edu (R.K.C.); 2 OU Genomics Core Facility, Ohio University, 1 Ohio University Drive Athens, Athens, OH 45701, USA; broach@ohio.edu; 3 Department of Biomedical Sciences, Ohio University Heritage College of Osteopathic Medicine, 1 Ohio University Drive Athens, Athens, OH 45701, USA; coschiga@ohio.edu; 4 Department of Biomedical Sciences and Pathobiology, Center for Molecular Medicine and Infectious Diseases, VA-MD College of Veterinary Medicine, Virginia Tech, 1410 Prices Fork Rd., Blacksburg, VA 24060, USA; caswellc@vt.edu

**Keywords:** *Shigella*, sRNA, pathogenesis, riboregulators, sibling, OmpC

## Abstract

Small regulatory RNAs (sRNAs) of *Shigella dysenteriae* and other pathogens are vital for the regulation of virulence-associated genes and processes. Here, we characterize RyfA1, one member of a sibling pair of sRNAs produced by *S. dysenteriae*. Unlike its nearly identical sibling molecule, RyfA2, predicted to be encoded almost exclusively by non-pathogenic species, the presence of a gene encoding RyfA1, or a RyfA1-like molecule, is strongly correlated with virulence in a variety of enteropathogens. In *S. dysenteriae*, the overproduction of RyfA1 negatively impacts the virulence-associated process of cell-to-cell spread as well as the expression of *ompC*, a gene encoding a major outer membrane protein important for the pathogenesis of *Shigella*. Interestingly, the production of RyfA1 is controlled by a second sRNA, here termed RyfB1, the first incidence of one regulatory small RNA controlling another in *S. dysenteriae* or any *Shigella* species.

## 1. Introduction

Species of the bacteria *Shigella* (*S. dysenteriae*, *S. sonnei*, *S. boydii*, and *S. flexneri*) are the causative agents of shigellosis, a highly infectious diarrheal disease in humans. Each year, an estimated 164 million people are infected by *Shigella*, resulting in 1.1 million deaths [1]. Devastatingly, children under the age of five in developing nations account for the majority of *Shigella*-associated deaths due to a lack of clean water, hydration, nutrition, and access to treatment [1]. *Shigella* infections, however, are not limited to developing countries; the Centers for Disease Control estimates 500,000 cases of shigellosis in the United States each year [2]. Worldwide prevalence of *Shigella* is vast, yet universally safe treatment and prevention are lacking [1]. These facts, together with the increasing rates of antibiotic resistance seen in *Shigella* species across the globe [3], highlight the relevance of these pathogens as continuing threats to human health, and thus the need for further investigation. Of particular importance are studies that reveal the molecular mechanism underlying the ability of *Shigella* species to survive within the human host and cause disease. These mechanisms must be fully characterized so that they can be specifically targeted by therapeutics, and by doing so, prevent or lessen the morbidity and mortality associated with shigellosis.

The basic pathogenesis pathway of *Shigella* is well characterized [4]. Infection of a new host occurs upon ingestion of *Shigella* during the consumption of contaminated food or water [1]. Once ingested, the pathogen transits the length of the gastrointestinal tract before reaching the colonic epithelium, the site where infection is initiated. *Shigella* cross from the lumen of the colon to the basolateral side of the colonic epithelium via uptake by microfold cells (M-cells) within the Peyer’s patches [5]. Following uptake by M-cells, the bacteria are presented to, and taken up by, macrophages. *Shigella* escape killing by the macrophages by inducing apoptosis of the phagocytic cells, a process that results in the release of the pathogen to the submucosa [5]. From here, *Shigella* utilize a Type III secretion system (TTSS) to orchestrate their own uptake into the nutrient-rich cytoplasm of the colonic epithelial cell [4,6]. Once within the eukaryotic cell, *Shigella* replicate and manipulate host cell actin to facilitate rapid inter- and intra-cellular spread [1,4,7,8,9]. The invasion of, replication within, and spread between cells of the colonic epithelium by *Shigella* provokes severe inflammation and destruction of the intestinal colonic epithelia, processes that result directly in the symptoms associated with shigellosis [1,4,7]. While studies characterizing the virulence-associated processes of *Shigella* pathogenesis are numerous, a complete understanding of these processes and the complex regulatory networks controlling them has not yet been achieved. Of particular interest is the growing evidence that RNA-mediated regulation plays a significant role in controlling virulence-associated processes in *Shigella* [10,11,12,13,14,15,16], a topic about which much remains to be learned.

It is increasingly recognized that regions within bacterial chromosomes previously assumed to be intergenic often encode riboregulators, RNA molecules that respond to a variety of signals and function to regulate the expression or activity of target genes or proteins [17,18,19]. Ribo-regulators can be classified into two major groups based on their location in relation to that of their target or targets. *Cis-*acting riboregulators are those that are located within the regulated transcript itself, while *trans*-acting ribo-regulators are encoded separately from their target. Small regulatory RNAs (sRNAs) that interact with target transcripts generated from gene(s) located distal to where the sRNA is encoded are classic examples of *trans*-acting ribo-regulators. While the understanding of bacterial sRNAs, their regulatory mechanisms, and their impact on critical cellular processes continues to grow exponentially, less well characterized are a sub-family of sRNAs termed sibling sRNAs [20]. So named due to their extensive similarly to each other, the relative function and significance of sibling sRNAs remain hotly debated [20]. What is known about sibling sRNAs is that many have important roles in regulating virulence-associated processes; however, the significance of having more than one near-identical copy of the regulator is often not completely understood.

To date, all riboregulators characterized in *Shigella* have been observed to function to influence virulence-associated processes, highlighting the importance of future studies of this class of regulators in *Shigella* and related pathogens [16]. Here we characterize RyfA1, one of a sibling pair of sRNAs in *S. dysenteriae*. While many bacterial species carry a single copy of *ryfA*, few contain sibling copies. With this study, it is demonstrated that RyfA1 influences the virulence-associated process of intracellular spread as well as the level of *ompC* transcript, a gene encoding a major outer-membrane protein associated with *Shigella* virulence. Additionally, it is demonstrated that RyfA1 production is controlled by RyfB1, a second sRNA encoded divergent to *ryfA1* that shares significant nucleic acid complementarity with RyfA1. Together, these sRNAs demonstrate the potential complex interplay between small transcripts in *S. dysenteriae*, an interaction that may allow for more precise and responsive regulation within virulence-associated networks. Disruption of these regulatory systems could be key to determining targets for therapeutic agents.

## 2. Materials and Methods

### 2.1. Growth Conditions

*Escherichia coli* K12 DH5α was grown in Luria–Bertani (LB) broth, tryptic soy broth (TSB), or agar at 37 °C. All strains of *S. dysenteriae* were grown in LB broth or TSB broth in a 200 rpm shaking incubator or cultured on tryptic soy broth agar (TSBA) plates with 0.01 wt/vol % Congo red at 37 °C unless otherwise noted. When needed to maintain selection for the presence of a plasmid, ampicillin was used at a concentration of 50 μg/mL.

### 2.2. Sequence Alignment and Structural Predictions of *RyfA1* and *RyfA2*

*E. coli* K12 MG1655 *ryfA* was used as a reference from the extensive National Center of Biotechnology Information database Basic Local Alignment Search [21] in order to find all other *ryfA* genes in bacteria whose genome are included. The genes *ryfA1* and *ryfA2* were aligned with each other using Clone Manager ver. 9 (Scientific & Educational Software, Denver, CO, USA). Structures of RyfA1 and RyfA2 were predicted using M-fold analyses from the mfold Web Server [22]. Species containing any *ryfA* gene were compared within the five nucleotide variable region using a program written in JavaScript (Brendan Eich, San Francisco, CA, USA) which scored gene likeness to *ryfA1* or *ryfA2* based on points. The greater the likeness score, the more similar. Supplemental figures comparing the likeness of all *ryfA* genes were generated using a CLC genomics workbench (QIAGEN, Valencia, CA, USA).

### 2.3. Cloning *ryfA1* and *ryfB1* into Expression Vectors

*ryfA1* was amplified from the *S. dysenteriae* chromosome [15] using specific primers containing *Mfe*I and *Hind*III restriction sites (Table S1). *ryfB1* was also amplified from the *S. dysenteriae* chromosome using primers containing *Mfe*I and *Sac*I restriction sites. Amplicons were run on an agarose gel and purified using the QIAquick gel extraction kit (QIAGEN) as per the protocol. Restriction enzymes *Mfe*I and *Hind*III from (New England Biolabs Inc., Ipswich, MA, USA) were used to digest the *ryfA1* containing amplicons as well as plasmid pQE2, an expression plasmid with an isopropyl β-D-1-thiogalactopyranoside (IPTG) inducible promoter (QIAGEN). Restriction enzymes *Mfe*I and *Sac*I from (New England Biolabs Inc.) were used to digest the *ryfB1* amplicon as well as pQE2. Both *ryfA*1 and *ryfB1* were subsequently ligated into pQE2 (QIAGEN) using T4 ligase (New England Biolabs Inc.) creating plasmids pRyfA1 and pRyfB1 respectively (Table S2). The resulting plasmids were introduced into competent *E. coli* K12 DH5α using heat shock transformation. Each plasmid was then extracted from DH5α using a QIAmini prep kit (QIAGEN) as per their instructions, sequence verified, and introduced into competent *S. dysenteriae* using electroporation.

### 2.4. Construction of GFP Translational Reporter Plasmid p5′UTR *RyfA1*

A double-stranded insert containing the putative 5′UTR of *ryfA1* (up to and including the translational start site) flanked by short single-stranded sequences compatible with overhangs generated by the activity of *Nsi*I and *Nhe*I restriction endonucleases was generated by oligo annealing. Once generated, the insert was ligated into plasmids pXG-10 [23] previously digested with restriction endonucleases *Nsi*I and *Nhe*I (New England Biolabs Inc.). Following ligation and introduction into competent *E. coli* DH5α by heat-shock transformation [24] the resulting translational reporter plasmid, termed p5′UTR RyfA1, was sequence verified using Sanger Sequencing (Ohio University Genomics Facility, Athens, OH, USA).

### 2.5. Western Blot

*E. coli* DH5α carrying pXG-0, pXG-1, or p5′UTR RyfA1 were grown to stationary phase at 37 °C. Using a ND-1000 spectrophotometer (NanoDrop Technologies, Wilmington, DE, USA), the optical density at 600 nm was measured and approximately 5 × 10^8^ bacterial cells were pelleted. The pelleted cells were suspended in 200 μL of a Laemmli protein dye (Bio-Rad, Hercules, CA, USA) supplemented with 5% 2-mercaptoethanol. Following suspension, the samples were boiled for 10 min and stored at −20 °C until further use. Fifteen microliters of each whole-cell protein preparation were separated using sodium dodecyl sulfate polyacrylamide gel electrophoresis (SDS-PAGE) on a 7.5% polyacrylamide gel. A polyvinylidene fluoride (PVDF) membrane was prepared by soaking in methanol for 10 min and rinsing with water three times. Proteins present in the polyacrylamide gel were then transferred to the prepared PVDF membrane. After the transfer, a 10% milk solution in phosphate buffered saline (PBST) was used to block the membrane overnight at 4 °C. Following blocking, anti-Gfp monoclonal immunoglobulin G (IgG) stabilized antibody preparation (Roche, Indianapolis, IN, USA) was diluted in PBST and 5% milk by 1:1000 and added to the membrane for incubation at 4 °C for one hour. The membrane was subsequently washed three times for 5 min each time in PBST and then incubated in PBST and a solution of 10% milk in PBST at 4 °C for 10 min. The secondary antibody (goat anti-mouse horseradish peroxidase (HRP) conjugated IgG (Bio-Rad)) was diluted 1:20,000 in PBST and 5% milk and incubated with the membrane for 1 h at 4 °C. Following incubation, the membrane was washed as above and the resulting signal visualized using the ImmuneStar WesternC reagents and a ChemiDoc XRS+ Imaging System (Bio-Rad).

### 2.6. RNA Isolation

Following growth under conditions indicated for each subsequent analysis (quantitative real-time polymerase chain reaction (qRT-PCR), Northern Blot) total RNA was isolated as follows. Bacterial cells present in 3 mL of culture were pelleted and lysed with the addition of 10% sodium dodecyl sulfate (SDS), and 3 M sodium acetate (pH of 5.2), followed by brief vortexing, then heating the cells and contents for 7 min at 90 °C. TRIzol (Thermo Fisher Scientific, Waltham, MA, USA) was subsequently added to the lysed cells, and all contents were transferred to a phase-lock tube (5 PRIME, Gaithersburg, MD, USA). Nucleic acid was isolated as per the factory protocol. RNA was precipitated overnight at −80 °C using 100% EtOH. The following day, RNA samples were centrifuged for 15 min on high at 4 °C and the supernatant discarded. Next, 1 mL of ice-cold 75% EtOH was added to the tube and the RNA pelleted again by centrifugation for 15 min on high at 4 °C. The supernatant was once again discarded and the RNA pellet dried, and subsequently rehydrated in 54 μL nuclease free water. Next, Turbo DNase (Ambion, Austin, TX, USA) was used according to the kit instructions to remove any DNA remaining in each RNA sample. To ensure that the RNA samples were free from DNA contamination, 1 μL of each was used as template in a screening PCR using primers for the conserved gene *sodB* and subsequently the amplicon was visualized on agarose gel. DNA-free RNA was measured using the Thermo Scientific Nanodrop 2000c spectrophotometer for concentration and quality based on 260/280 and 260/230 ratios.

### 2.7. Northern Blot

Following growth at 37 °C in a shaking incubator, RNA was isolated as described above. Ten micrograms of RNA were run on a 10% polyacrylamide gel with 7 M urea and 1× TBE (89 mM boric acid, 89 mM Tris, and 2 mM EDTA). A Low Molecular Weight DNA Ladder (New England BioLabs, Inc.) was labeled with [ɣ-32P]-ATP using polynucleotide kinase and run on the same gel as the RNA sample. RNA and ladder were separated at 150 volts in 1× TBE buffer. Nucleic acids were then transferred to a Hybond™-N+ membrane (GE Healthcare, Piscataway, NJ, USA) at 50 volts for 2 h. The samples and ladder were then crosslinked using UV to the membrane. Probes were labeled by polynucleotide kinase with [ɣ-32P]-ATP. The membrane was pre-hybridized in ULTRAhyb^®^-Oligo Buffer (Ambion) at 42 °C for at least 2 h in a rotating incubator. Radio-labeled probes were then added to the pre-hybridized membrane and allowed to hybridize overnight at 42 °C in a rotating incubator. The membrane was washed with 2× saline-sodium citrate (SSC) (300 mM NaCl, and 30 mM NaC6H5O7) + 0.1% SDS, 1× SSC + 0.1% SDS, and 0.5× SSC + 0.1% SDS for 30 min at 42 °C in a rotating incubator. The membranes were then exposed to film and visualized by autoradiography.

### 2.8. Quantitative Real-Time PCR Analysis

Total RNA was isolated from triplicate samples using methods described above following growth of each strain to the mid-logarithmic phase in LB supplemented with 20 μM IPTG, 50 μg/μL ampicillin and 0.01% deoxycholic acid. To generate complementary DNA (cDNA) for analysis, iScript (Bio-Rad) was used following the manufacturer’s instructions. cDNA samples were diluted 1 to 10 and used as templates in real-time PCR analyses with TaqMan probe chemistries for analysis of *ryfA1*, *ryfA2,* and *ryfB2*, or Sybrgreen and iQ Supermix (Bio-Rad) for analysis of *ompC*, as per the instructions. All samples were amplified and analyzed in a Bio-Rad C1000™ Thermal Cycler with a CFX96 Real-Time System under standard reaction conditions optimized for each primer set (Table S1). Fold changes were calculated using the ΔΔCt method [25] with target amounts normalized to those of the housekeeping gene *rrsA* in each sample and expressed relative to a selected control sample.

### 2.9. Plaque Assay

Henle cells were cultured at 37 °C in an atmospheric condition of 5% CO_2_ to 80% confluency in six-well polystyrene tissue culture plates (Corning Inc. Costar, Corning, NY, USA) in the presence of Henle Cell Media (composed of Gibco Minimal Essential Media) (Invitrogen Corp., Carlsbad, CA, USA), supplemented with 10% Fetal Bovine Serum (FBS), 2 mM glutamine, and 1× non-essential amino acids (Lonza, Basel, Switzerland).

Plaque assays were performed as described previously by Oaks et al. 1985 [26] with minor modifications. *S. dysenteriae* strains were cultured on TSBA with Congo red and 50 μg/mL ampicillin. Three colonies from each strain were used to inoculate separate 3-mL cultures of TSB and 50 μg/mL ampicillin, which were subsequently grown overnight in a shaking incubator at 30 °C. One hundred microliters of each overnight culture were used to inoculate 3 mL of fresh TSB supplemented with 50 μg/mL ampicillin, 20 μM IPTG, and 0.01% deoxycholate (DOC). Strains were cultured at 37 °C in a shaking incubator to the mid-logarithmic phase of growth prior to the addition of 10^4^ bacteria from each culture into a separate well of a six-well polystyrene tissue culture plate containing a monolayer of Henle cells at approximately 80% confluency, 2 mL of Henle media, 50 μg/mL ampicillin, and 20 μM IPTG. Following inoculation with the bacteria, the tissue culture plates were spun for 10 min in a Beckman Coulter Allegra 25R centrifuge (Brea, California, USA) at 600× *g* at room temperature and then incubated for 90 min in a 37 °C incubator under 5% CO_2_ atmospheric conditions. Following incubation, Henle media was suctioned off and to the monolayer a wash containing 2 mL of fresh Henle media supplemented with 50 μg/mL ampicillin, 20 μg/mL gentamicin, 0.3% glucose, and 20 μM IPTG was added. Following incubation for 72 h at 37 °C and in 5% CO_2_ atmospheric conditions the Henle cell monolayers and bacteria were stained with Giemsa–Wright stain (Camco, Ft. Lauderdale, FL, USA) and washed with double distilled water twice. Plaques were analyzed for number and size.

### 2.10. Spread Assay

Spread assays were performed as a modified version of the plaque assay. *S. dysenteriae* strains were grown as described above but to near confluency. Once the bacteria reached mid-logarithmic growth phase, 1 × 10^4^ *Shigella* cells were used to infect a near confluent monolayer of Henle cells in Henle media containing 50 μg/mL ampicillin and 20 μM IPTG. Plates were centrifuged as described above. Following centrifugation, plates were subsequently incubated for 60 min at 37 °C at an atmospheric condition of 5% CO_2_. Next, Henle cells were washed with 2 mL of Henle media, and then an overlay of Henle Media containing 0.3% glucose, 50 μg/mL ampicillin, 20 μM IPTG, and 20 μg/mL of gentamicin was added. Plates were incubated for 6 h at 37 °C under 5% CO_2_ atmospheric conditions. After this incubation, Wright–Giemsa stain (Camco, Ft. Lauderdale, FL, USA) was used to stain the plates. The plates were washed with distilled water following staining and air-dried. One hundred Henle cells containing ≥3 and that were in contact with at least two other Henle cells within the monolayer were selected for scoring. Spread was scored if one or more of the Henle cells in physical contact with the first also contained bacteria.

### 2.11. Invasion Assay

Invasion assays were performed as a modification to plaque assays. *S. dysenteriae* strains were grown as described above. Once reaching mid-logarithmic growth phase, 2 × 10^8^ bacteria were used to infect a 60% confluent monolayer of Henle cells, as detailed above. Each well contained Henle media, and was supplemented with 50 μg/mL ampicillin and 20 μM IPTG. Plates were centrifuged as described above and incubated for 30 min at 37 °C at 5% atmospheric CO_2_. Following incubation, plates were washed with 2 mL of Henle media, and then covered in Henle cell media containing 0.3% glucose, 50 μg/mL ampicillin, 20 μM IPTG, and 20 μg/mL gentamicin. Plates were incubated for 90 min at 37 °C under 5% CO_2_ atmospheric conditions. Following incubation, plates were stained with Wright–Giemsa stain (Camco, Ft. Lauderdale, FL, USA). Next, plates were washed with distilled water, air-dried, and invaded Henle cells were counted. Henle cells that contained ≥3 bacteria and were physically isolated from other Henle cells were scored as invaded.

### 2.12. Next-Generation Sequencing

The following RNA isolation protocol was adapted from Carroll et al. [27] with minor modifications. *S. dysenteriae* strains carrying either the empty vector (pQE2) or the *ryfA1* expression plasmid (pryfA1) (Table S2) were cultured to the mid-logarithmic phase of growth at 37 °C in 3 mL LB containing 50 μg/mL ampicillin and 0.01% DOC and 20 μM IPTG. Next, 750 μL of RNA preserving solution (95% EtOH, 5% phenol) were added to each tube. Bacterial cells present in each sample were pelleted and the supernatant discarded. To isolate total RNA, QIAGEN RNEasy kit (QIAGEN) was used as per instructions. Immediately following, nucleic acid was treated with Turbo DNAse (Ambion). RNA was checked for purity and concentration using Agilent 2100 Bioanalyzer on an RNA Nano 6000 chip. Ribosomal RNA was depleted from the samples using Ribozero (Illumina, San Diego, CA, USA) and MicrobExpress (Thermo Fisher Scientific) as per their respective instructions. RNA was ethanol precipitated in the presence of 3 M sodium acetate and 1 μg/μL RNA-grade glycogen (Thermo Fisher Scientific) overnight at −80 °C. The following day, samples were centrifuged at 4 °C for 30 min. The supernatant was discarded and 1 mL of ice cold 70% EtOH was added. Samples were spun in a microcentrifuge on high for 10 min at 4 °C. Again, the supernatant was discarded and the RNA was allowed to air dry for 5 min. Each RNA pellet was suspended in 20 μL of nuclease-free water and analyzed for purity and concentration as above. RNA-seq was performed using an Ion Torrent Next PGM and 200 bp read chemistry and a 318 sequencing chip at the Ohio University Genomics Core Facility. Data was analyzed using CLC Genomics Workbench Version 8 (QIAGEN).

The RNAseq data files have been deposited in GEO under accession number GSE87727.

## 3. Results

### 3.1. Twin Copies of *ryfA* Are Produced by *Shigella dysenteriae*

**Figure 1:**
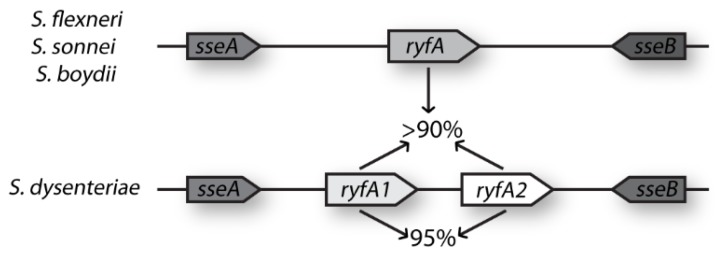
*S. dysenteriae* encodes for two nearly identical RyfA molecules, sibling small regulatory RNAs (sRNAs) here designated RyfA1 and RyfA2. RyfA1 and RyfA2 represent putative sibling sRNAs 95% identical to one another and with greater than 90% nucleic acid identity to the singlet RyfA encoded at the same chromosomal location in all other species of *Shigella* as well as several isolates of *E. coli*.

**Figure 2:**
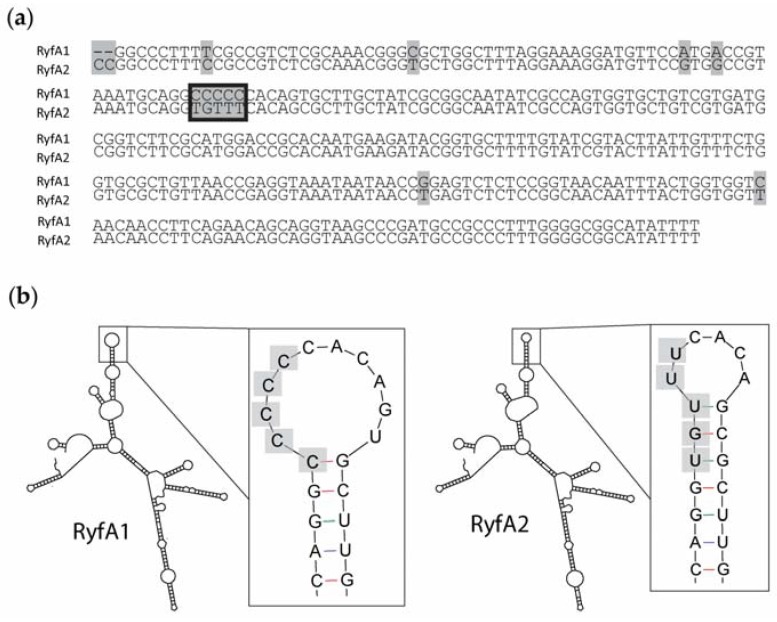
RyfA1 and RyfA2 structure and sequence. (**a**) The predicted genes *ryfA1* and *ryfA2* were aligned using Clone Manager version 9. The genes encoding the sibling RyfA molecules are 95% identical in *S. dysenteriae*. Nucleotide differences between *ryfA1* and *ryfA2* are highlighted with light gray rectangles and the identified five nucleotide “variable region” of each is boxed in black. (**b**) The mfold Web Server [22] was used to predict the structures of RyfA1 and RyfA2. Although the predicted structure between each sibling RyfA is nearly identical, the stem loops outlined in the black boxes vary between the two molecules. The observed structural differences between RyfA1 and RyfA2 result from the varied nucleic acid sequences within the five nucleotide variable region (as indicated by the gray boxes) identified upon a comparison of each *ryfA* gene.

Originally identified by in silico*-*based genomic analyses designed to identify genes encoding previously uncharacterized sRNA molecules, the putative sRNA RyfA was predicted to be encoded by *Shigella flexneri* and several strains of *Escherichia coli* [28]. Further in silico analyses revealed that *ryfA* is present in other species of *Shigella* (*S. boydii*, *S. sonnei*, and *S. dysenteriae*) (Figures S1 and S2). Of note, two genes predicted to encode nearly identical sibling sRNAs, here designated *ryfA1* and *ryfA2*, were found to exist in tandem on the *S. dysenteriae* chromosome (Figure 1). *ryfA1* and *ryfA2* share 95% identity at the nucleic acid level, and each has greater than 90% sequence identity to the singlet *ryfA* from other species (Figure 1). While several single-nucleotide alterations exist between *ryfA1* and *ryfA2*, the largest run of nucleotide dissimilarities occurs in a five-nucleotide stretch, here termed the variable region (Figure 2a). This five-nucleotide variable region is not limited to *ryfA1* and *ryfA2* in *S. dysenteriae*; indeed, across all isolates containing *ryfA*, the least conserved region is the variable region (Figure S1). In *S. dysenteriae* the variable region influences the predicted structure of RyfA1 and RyfA2 in a subtle but potentially important way. Specifically, while otherwise identical, the predicted structure of RyfA1 and RyfA2 varies within a single stem-loop structure containing the sequences from within the variable region of each (Figure 2b) [22]. Given the significance of single-stranded regions within sRNAs in mediating specific interactions between the sRNA and its regulatory target(s), the variation predicted between RyfA1 and RyfA raises the possibility that these two sRNAs may have unique regulatory targets, and thus unique functions, in vivo.

**Figure 3:**
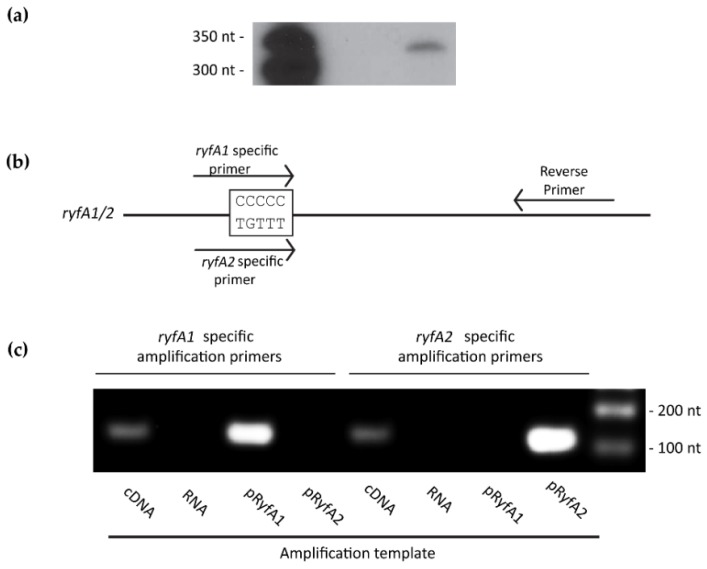
*S. dysenteriae* produces both RyA1 and RyfA2. (**a**) Northern blot analysis using total RNA isolated from wild-type *S. dysenteriae* following growth to the mid-logarithmic phase at 37 °C and a radio-labeled probe specific to sequences conserved between RyfA1 and RyfA2. The predicted sizes of RyfA1 and RyfA2 are 303 and 305 nt, respectively. (**b**) Schematic depicting the location and sequence specificity of the amplification primers used in the reverse transcriptase analysis. Each forward primer overlaps the five-nucleotide variable region of RyfA1 or RyfA2, thus providing specificity of amplification. (**c**) Reverse transcriptase PCR demonstrating that both RyfA1 and RyfA2 are produced by wild-type *S. dysenteriae* under the conditions tested. Using the *ryfA1* specific forward primer, amplification occurs when complementary DNA (cDNA) generated from *S. dysenteriae* or a plasmid carrying *ryfA1* is used as a template (pRyfA1), but not when a plasmid carrying *ryfA2* is used as a template (pRyfA2). Similarly, using the *ryfA2* specific forward primer, amplification occurs when cDNA generated from *S. dysenteriae* or a plasmid carrying *ryfA2* is used as a template, but not when a plasmid carrying *ryfA2* is used as a template. The RNA used to generate the cDNA amplification template is used itself as a template with each primer set to ensure that the sample is free from DNA contamination.

The first step in characterizing RyfA1 and RyfA2 from *S. dysenteriae* was to determine if the bacterium produces one or both of these putative molecules. To this end, a northern blot was performed using a probe specific for a region conserved between RyfA1 and RyfA2; sRNAs predicted to be 303 nt and 305 nt in length, respectively. A single band of approximately 300 nt was detected in the northern blot analysis, confirming that *S. dysenteriae* produces at least one RyfA molecule (Figure 3a). To investigate the presence of both RyfA1 and RyfA2 in wild-type *S. dysenteriae*, RT-PCR analyses were performed. Specifically, cDNA was generated from total RNA isolated from wild-type *S. dysenteriae* and used as template in amplification reactions pairing a conserved reverse primer with a forward primer specific to the variable region of either RyfA1 or RyfA2 (Figure 3b). Plasmids containing either *ryfA1* or *ryfA2* were used as control templates to ensure the *ryfA1* specific primers only amplified *ryfA1* and not *ryfA2*, and vice versa. Results from the RT-PCR analyses demonstrate that amplification by each primer set is specific to the corresponding RyfA molecule and, importantly, that wild-type *S. dysenteriae* produces both RyfA1 and RyfA2 (Figure 3c).

### 3.2. The Presence of a *ryfA1*-Like Gene Is Associated with Pathogenicity

**Figure 4:**
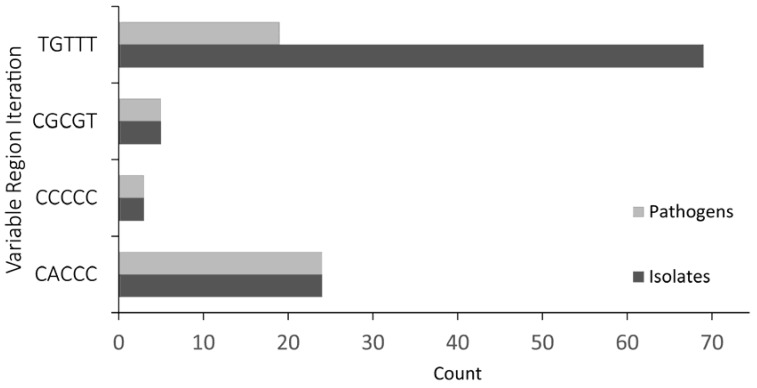
Bacterial isolates that encode for a RyfA1-like molecule are enteropathogens. All bacterial isolates whose genome is sequenced and available on the National Center for Biotechnology Information (NCBI) [21], were evaluated for the presence of *ryfA*. Next all identified species were grouped based on the similarity of nucleic acid sequence within the *ryfA* five-nucleotide variable region. Four different iterations of the variable region were found, TGTTT, CGCGT, CCCCC, and CACCC. Three of the four variable region iterations contained higher GC content and were termed “*ryfA1*-like”. The number of isolates containing each RyfA iteration as well as the number of those with each group that are pathogenic are indicated.

In silico analysis of all sequenced bacterial isolates available in the National Center for Biotechnology Information [21] and ShiBASE [29] databases was carried out in order to identify those that carry one or more *ryfA* gene. Interestingly, *ryfA* was identified only in *Shigella* and *Escherichia* isolates. Next, using an in-house program written in JavaScript, the identified *ryfA* genes were scored and grouped based on likeness within the five-nucleotide variable region. Our analysis revealed that four iterations of the five-nucleotide variable region exist in *Shigella* and *Escherichia*, three versions being grouped as “*ryfA1*-like” by containing higher GC content (CACCC, CCCCC, and CGCGT) and a single *ryfA2* version with higher T content (TGTTT) (Figure 4). Strikingly, of the isolates that contain a *ryfA* gene, all that encode a “RyfA1-like” molecule are pathogenic (Figure 4), and more specifically enteropathogenic. With a few exceptions, bacterial isolates that carry a *ryfA2* gene are either non-pathogenic or uropathogenic. Given the observed link between the presence of a *ryfA1-*like gene and pathogenicity, *S. dysenteriae* RyfA1 was selected for further investigation.

### 3.3. *ryfA1* Impacts Virulence in *Shigella dysenteriae*

#### 3.3.1. Overproduction of RyfA1 Does Not Significantly Impact the Growth of *S. dysenteriae*

**Figure 5:**
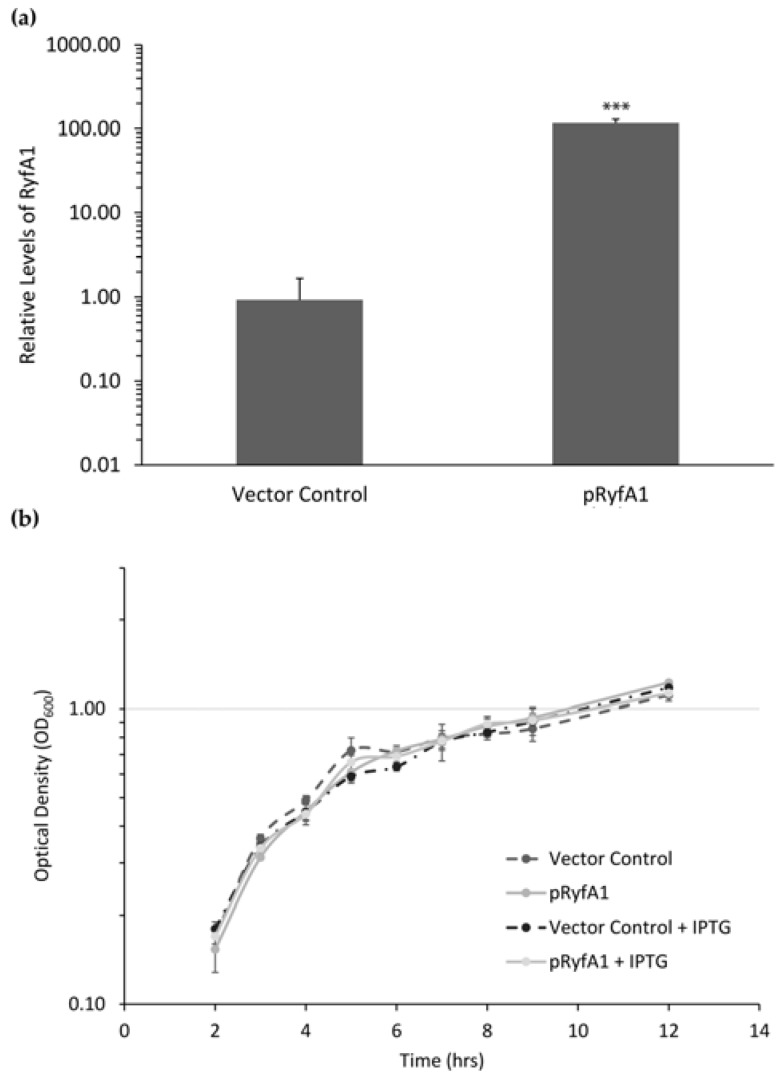
Overproduction of RyfA1 has no significant impact on the growth of *S. dysenteriae*. An isopropyl β-D-1-thiogalactopyranoside (IPTG) inducible *ryfA1* expression plasmid was created (pRyfA1) and introduced into *S. dysenteriae*. (**a**) Quantitative real-time PCR (qRT-PCR) analysis of the relative levels of RyfA1 present in *S. dysenteriae* carrying pRyfA1 or the pQE2 vector control following the growth of each strain in the presence of IPTG. RyfA1 levels were calculated using the ΔΔCt method [25] in which they are normalized to the level of *rrsA* measured in each sample and expressed relative to the level of RyfA measured in a single vector control sample. All data are the average of biological triplicate analyses and error bars represent one standard deviation. *** denotes a statistically significant difference with *p* < 0.0001. (**b**) Growth analysis of *S. dysenteriae* carrying the pQE2 vector control was compared to that of the strain carrying pRyfA1 under both non-inducing (0 μM IPTG) and inducing (20 μM IPTG) conditions. No significant differences were observed between the growth of *S. dysenteriae* carrying pRyfA1 or the vector control under either condition tested.

To characterize the function of RyfA1 in *S. dysenteriae*, *ryfA1* was amplified and ligated into an expression plasmid such that production of the RyfA1 molecule was placed under the control of an IPTG inducible promoter (pRyfA1). To ensure that RyfA1 is predictably produced from the pRyfA1 plasmid, qRT-PCR analyses were carried out using a primer probe set designed to distinguish *ryfA1* from *ryfA2* by binding of the probe over the five-nucleotide variable region. The growth of *S. dysenteriae* carrying pRyfA1 in the presence of 20 μM IPTG results in a 100-fold increase in the relative levels of RyfA1 as compared to those measured in the strain carrying the empty vector control (pQE2) grown under identical conditions (Figure 5a). Increased levels of RyfA1 have no significant effect on the growth of *S. dysenteriae* as determined by growth analysis of the strain carrying pRyfA1 or the vector control in the presence of 20 μM IPTG (Figure 5b).

#### 3.3.2. Overproduction of RyfA1 Leads to Inhibition of Cell-to-Cell Spread by *Shigella dysenteriae*

**Figure 6:**
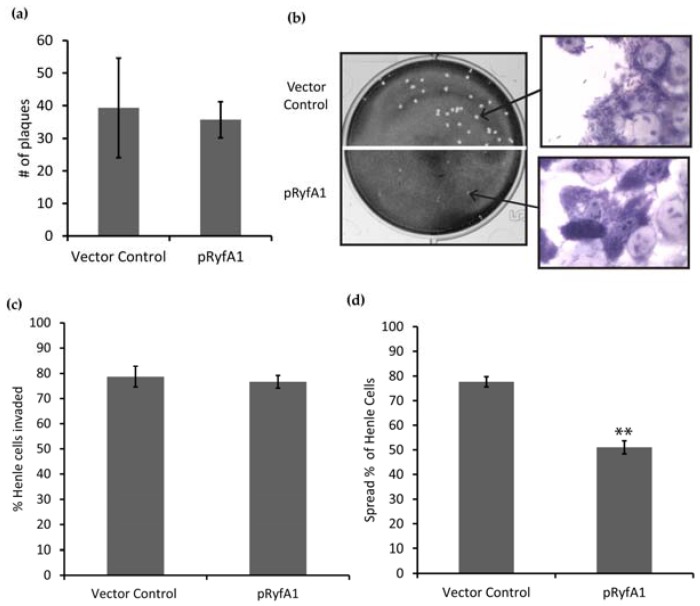
RyfA1 overproduction inhibits cell to cell spread by *S. dysenteriae*. (**a**) No significant difference was observed in the number of plaques formed between the vector control and pRyfA1. (**b**) Representative image of plaques formed by *S. dysenteriae* carrying either the pQE2 vector control or the *ryfA1* producing plasmid pRyfA1 cultured and maintained under inducing conditions. The magnified image is of Henle cells (larger light purple cells) and bacterial cells (dark purple smaller cells) surrounding the plaque formed by the indicated strain. Quantification of the ability of *S. dysenteriae* carrying the RyfA1-producing plasmid pRyfA1 or the vector control to invade Henle cells (**c**) and to spread between Henle cells within a subconfluent monolayer (**d**). All assays were carried out following growth of the indicated strain in the presence of IPTG, and the inducer was maintained throughout the analyses. Data presented in panels (**a**), (**c**), and (**d**) are the average of biological triplicate analyses and error bars represent one standard deviation. ** denotes a statistically significant difference with *p* < 0.001.

The ability of RyfA1 to impact virulence processes in *S. dysenteriae* was investigated using a series of in vitro tissue culture-based analyses. The first assays completed were plaque assays that measure the cumulative ability of the bacterium to invade, replicate within, and spread between eukaryotic cells within a monolayer. Maintaining the strains under inducing conditions to ensure increased production of RyfA1 from the pRyfA1 plasmid, the ability of *S. dysenteriae* carrying pRyfA1 to form plaques within a monolayer of eukaryotic cells was compared to that of the strain carrying the vector control. Interestingly, *S. dysenteriae* carrying pRyfA1 formed an equivalent number of plaques to those formed by the strain carrying the vector control, yet the plaques formed were dramatically smaller (Figure 6a,b). These data indicate that increased production of RyfA1 inhibits plaque formation and, specifically, based on the conserved number and decreased size of the plaques, predicts that it is the process of cell-to-cell spread that is inhibited.

To determine which step of plaque formation is specifically influenced by RyfA1, in vitro invasion assays and spread assays were performed. For each of these assays, wild-type *S. dysenteriae* carrying either the pQE2 vector control or the RyfA1 producing plasmid pRyfA1 were cultured under inducing conditions. While *S. dysenteriae* expressing RyfA1 from pRyfA1 invaded eukaryotic cells with the same efficiency as the strain carrying the vector control grown (Figure 6c), the efficiency of cell-to-cell spread was significantly lower (Figure 6d). Taken together, these data clearly demonstrate that increased production of RyfA1 limits plaque formation by inhibiting the ability of *S. dysenteriae* to efficiently spread from one cell to the next, a process essential for the full virulence of this bacterial pathogen.

### 3.4. RyfA1 Overproduction Results in Elimination of ompC, a Transcript Encoding a Major Outer Membrane Protein

**Figure 7:**
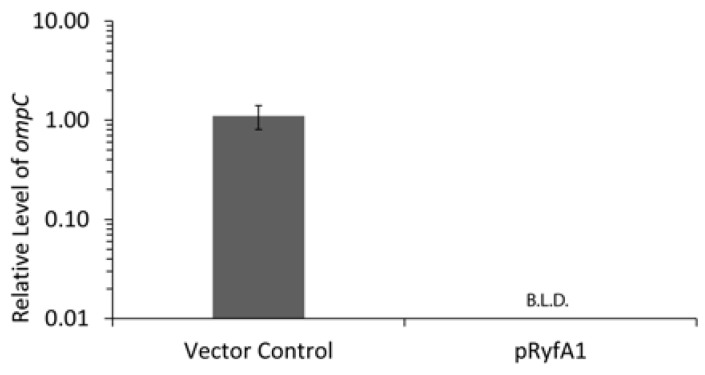
Increased production of RyfA1 results in undetectable levels of *ompC* transcript. qRT-PCR measuring the relative abundance of *ompC* transcript levels in *S. dysenteriae* carrying the RyfA1 producing plasmid pRyfA1 or the vector control following growth in the presence of IPTG. Using the ΔΔCt method [25], *ompC* levels are normalized to that of *rrsA* in each sample and are expressed relative to a single vector control sample. Data presented are the average of analyses completed in biological triplicate with error bars representing one standard deviation. B.L.D. indicates that the target transcript was below the level of reliable detection.

Inhibition of cell-to-cell spread in *S. dysenteriae* can result from misregulation or malfunction of any number or variety of molecular factors. RNA-seq analyses were performed as a means to identify those genes for which transcript levels change significantly as a result of increased RyfA1 production. When the transcriptome of wild-type *S. dysenteriae* producing RyfA1 from pRyfA1 was compared to that of the strain carrying the empty vector, the single largest fold change in transcript level (≈7000 fold) was observed for *ompC*, a transcript encoding the major outer membrane protein C (OmpC). The negative impact of RyfA1 production on *ompC* levels was confirmed using qRT-PCR (Figure 7). Interestingly, OmpC has been shown to influence *Shigella* virulence. Specifically, inactivation of *ompC* in *S. flexneri* has been shown to inhibit cell-to-cell spread by the pathogen, as measured using in vitro tissue- culture-based analyses [30]. Together, these data demonstrate that increased production of RyfA1 in wild-type *S. dysenteriae* results in undetectable levels of *ompC* and a phenotype that phenocopies that of *S. flexneri* lacking *ompC*, an inability to spread from one cell to the next within a monolayer of human epithelial cells.

### 3.5. Regulation of *ryfA1* by *ryfB1*

**Figure 8:**
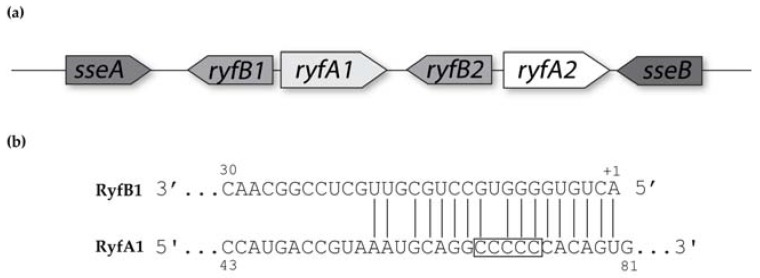

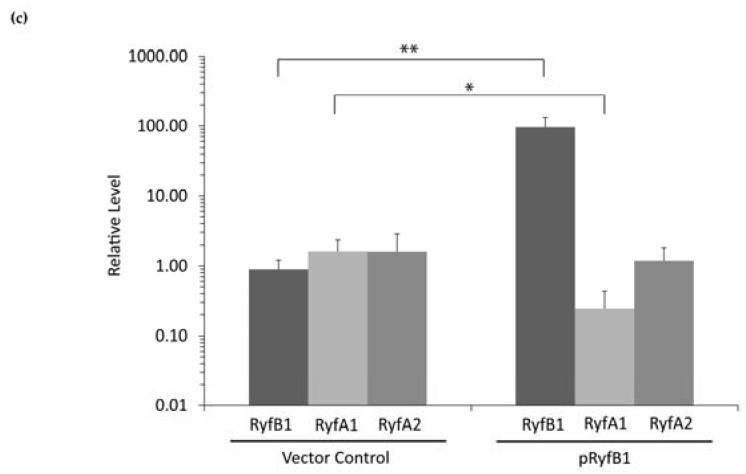
RyfB1 specifically inhibits RyfA1. (**a**) Next-generation sequencing revealed ≈100 nt long transcripts immediately preceding, and divergent to, *ryfA1* and *ryfA2.* The gene preceding *ryfA1* has been termed *ryfB1,* while that preceding *ryfA2* has been termed *ryfB2*. (**b**) In silico analyses of RyfB1 revealed a region, in which 16 of 18 nucleotides share complementarity to sequences within RyfA1. Of note, this region of complementarity between RyfA1 and RyfB1 overlaps the five-nucleotide variable region of the latter, indicated by the black box. Numbers indicate the base location relative to the 5′ end of each molecule (+1). (**c**) qRT-PCR analyses of the relative levels of RyfB1, RyfA1, and RyfA2 in *S. dysenteriae* carrying the RyfB1-producing plasmid pRyfB1 or the vector control following growth of both strains under inducing conditions. The relative abundance of each target was calculated using the ΔΔCt method [25], in which target transcript levels are normalized to the level of *rrsA* present in each sample and are expressed relative to the level in that target in a single vector control sample. Data are the average of biological triplicates and errors bars indicate one standard deviation. * denotes a significant difference with *p* < 0.01 while ** denotes a significant difference with *p* < 0.001.

The RNA-seq analyses provided unique insight into the *ryfA1*/*ryfA2* locus on the *S. dysenteriae* chromosome. Specifically, a small transcript (≈100 nt in length) was detected upstream of, and encoded divergently to, each *ryfA* gene (Figure 8a). These small transcripts are likely to encode putative sRNAs, due to a lack of an ATG start site or a discernable ribosomal binding site. Designated *ryfB1* and *ryfB2*, these putative sRNAs share 60% of nucleic acid with each other. Despite the fact that the coding regions for *ryfA1* and *ryfB1* do not overlap, in silico analysis demonstrated that the transcripts share robust nucleic acid complementarity with each other. Specifically, within an 18-nucleotide-long region, 16 nucleotides demonstrate complementarity between the *ryfA1* and *ryfB1* transcripts (Figure 8b). It is noteworthy that the region of complementarity between RyfA1 and RyfB1 overlapped the variable region of RyfA1. Together these observations lead to the testable hypothesis that *ryfB1* encodes an sRNA that functions to specifically regulate RyfA1.

To experimentally determine if RyfB1 influences RyfA1 levels, and if this regulation is specific, an expression plasmid was generated in which *ryfB1* is cloned under the control of an IPTG inducible plasmid (pRyfB1) and introduced into wild-type *S. dysenteriae*. Following the growth under inducing conditions, quantitative RT-PCR analysis was used to measure the relative levels of RyfA1, RyfA2, and RyfB1 in *S. dysenteriae* carrying either pRyfB1 or the vector control. The obtained data demonstrate that increased levels of RyfB1 result in a significant decrease in the relative amounts of RyfA1 (Figure 8c). The specificity of RyfB1 regulation on RyfA1 is demonstrated by the fact that increased production of RyfB1 has no significant effect on the relative abundance of RyfA2 (Figure 8c).

### 3.6. RyfA1 Does Not Encode a Small Protein under the Conditions Tested

**Figure 9:**
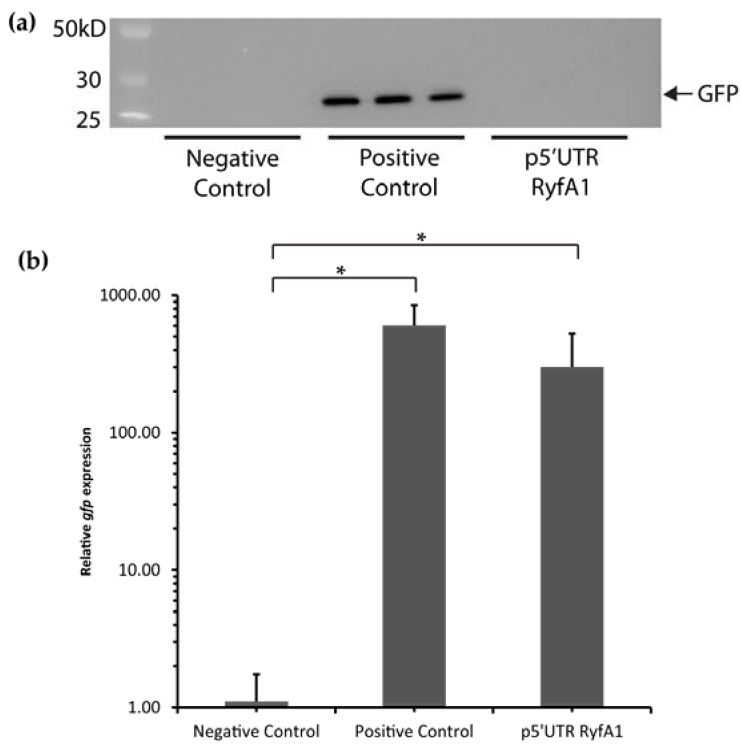
Sequences within the *ryfA1* transcript do not mediate translation initiation. (**a**) Western blot analysis was used to detect GFP production in *E. coli* carrying a negative control plasmid (pXG-0), the experimental plasmid p5′UTR RyfA1, or a positive control plasmid (pXG-1). Under the conditions tested, no GFP was produced in the strain carrying p5′UTR RyfA1, indicating that the cloned sequences do not support translation initiation, and thus that the *ryfA1* transcript likely does not encode a small peptide. (**b**) qRT-PCR was used to ensure that the same triplicate samples used to detect GFP protein were producing *gfp* transcript. Strains carrying either the positive control or the p5′UTR RyfA1 plasmid produce significantly more transcript than the negative control. Error bars represent one standard deviation. * indicates a significant difference (*p* < 0.05). Relative levels were calculated using the ΔΔCt method [25] where *gfp* levels are normalized to the amount of *rrsA* present in each sample and are expressed relative to that present in a single pXG-10 sample.

The genetic arrangement, relative size, and nucleic acid complementarity between *ryfA1* and *ryfB1*, as well as the ability of RyfB1 to influence steady-state levels of RyfA1 (Figure 8b), is highly reminiscent of a Type I toxin-antitoxin locus, especially that of the *zorO-orzO* systems of *E. coli* O157:H7 [31,32]. In the case of the *E. coli* *zorO-orzO* toxin-antitoxin system, the larger *zorO* transcript encodes a small peptide that is toxic to *E. coli* O157:H7, and the sRNA OrzO inhibits production of that toxin [31,33]. The regulation of *zorO* by OrzO is dependent on the 18 nucleotides of complementarity between the two transcripts [31]. In order to test if the *ryfB1-ryfA1* pair encode a classical Type I toxin-antitoxin system, we first searched for putative ribosomal binding sites and potential start codons within each transcript by in silico analyses using Clone Manager Version 9. Within *ryfA1*, a putative ribosomal binding site and corresponding ATG start codon were located that, if functional, would be predicted to result in the synthesis of a small protein composed of 30 amino acids. To test whether or not a protein could be made from the *ryfA1* transcript, the putative 5′- untranslated region, up to and including the identified predicted translational start of the gene, was ligated into plasmid pXG-10 (Table S2) between a constitutive promoter and in frame with the *gfp* reporter gene lacking its own ribosomal binding and translational start sites. If the cloned sequence from *ryfA1* is capable of mediating ribosomal binding and translation initiation under the conditions tested, GFP would be produced, a prediction that was tested using Western Blot analysis. Specifically, total protein was extracted from triplicate strains of *E. coli* DH5α containing either p5′UTR RyfA1, or the positive/negative control plasmids. No GFP protein was detected in the strains carrying p5′UTR RyfA1 (Figure 9a). To ensure *gfp* transcript was being produced, qRT-PCR was performed on the sample triplicate samples (Figure 9b). Significantly more *gfp* transcript was detected in the positive control and p5′UTR RyfA1 than in the negative control. Together, these data suggest that, under the conditions tested, no protein is generated from the *ryfA1* transcript.

**Figure 10:**
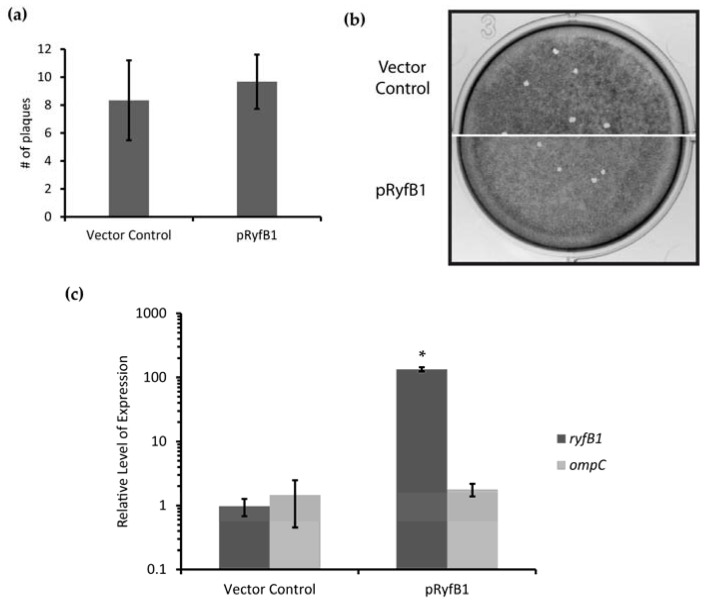
Overproduction of RyfB1 does not impact plaque formation of *S. dysenteriae*. (**a**) Overproduction of RyfB1 by *S. dysenteriae* did not alter the number of plaques as compared to a vector control. (**b**) A monolayer of Henle cells was infected with *S. dysenteriae* containing a vector control or overproducing RyfB1 (pRyfB1). Overproduction of RyfB1 did not alter the plaque phenotype as from the vector control. (**c**) qRT-PCR was used to measure expression of RyfB1 and *ompC*. Significant overproduction of RyfB1 did not impact the relative levels of *ompC* transcript. * denotes a significant difference where *p* < 0.001. The relative levels of RyfB1 and *ompC* were calculated using the ΔΔCt method [25]. The housekeeping gene, *rrsA*, was used as an internal control.

Increased production of RyfA1 results in both a decrease in the steady-state level of *ompC* and an inhibition of the ability of *S. dysenteriae* to form plaques in a monolayer of human epithelium cells (Figure 6 and Figure 7). The finding that RyfB1 production decreased the steady-state levels of RyfA1 (Figure 8) allows for the evaluation of the impact of reduced RyfA1 levels on the relative abundance of *ompC* and on the ability of *S. dysenteriae* to form plaques in a monolayer of human epithelium cells. To this end, first, the ability of *S. dysenteriae* overexpressing RyfB1 from the pQE plasmid to form plaques in a Henle cell monolayer was compared to that of the strain carrying the vector control. No significant differences in plaque formation were observed between *S. dysenteriae* containing the vector control and the strain overexpressing pRyfB1 (Figure 10a,b). Next, using the same set of strains, the impact of RyfB1 overproduction on the steady-state levels of *ompC* was directly investigated using qRT-PCR. As would be predicted, an increase in RyfB1 levels had no significant effect, either positive or negative, on the relative amount of *ompC* as compared to levels measured in the strain carrying the vector control (Figure 10c).

## 4. Discussion

The presented studies demonstrate that, unlike related species, *S. dysenteriae* encodes and produces two RyfA molecules, RyfA1 and RyfA2. Consistent with the positive correlation between the presence of a RyfA1-like molecule and virulence, *S. dysenteriae* RyfA1 has now been shown to influence virulence-associated processes in this pathogen. Specifically, increased production of RyfA1 inhibits the ability of *S. dysenteriae* to spread from cell to cell within a monolayer of human epithelial cells, and results in near-elimination of *ompC* transcript, a porin protein important for cell-to-cell spread in *S. flexneri* [30]. Furthermore, RNAseq analysis revealed the presence of a short divergently encoded transcript immediately upstream of *ryfA1*. The transcribed product of this newly identified gene, designated RyfB1, shares robust complementarity to RyfA1 and when overproduced results in a significant decrease in RyfA1 levels. Taken together, these data demonstrate that RyfA1 impacts cell-to-cell spread by *S. dysenteriae*, a process that is crucial for the pathogenesis of the pathogen. Furthermore, RyfA1 is now implicated in a complex regulatory network involving *ompC* and an additional sRNA RyfB1. As such, both RyfA1 and RyfB1 are now implicated in the regulation of virulence-associated processes in *S. dysenteriae*, and thus represent two newly identified virulence factors.

The specific regulatory mechanisms and action of RyfB1 and RyfA1 have yet to be elucidated. However, based on the data presented above, some predictions can be made. RyfA1 and RyfB1 share 17 nucleotides of complementarity, a finding that leads to the model that RyfB1 influences RyfA1 levels via a direct interaction between these two molecules. Of the 17 nucleotides with complementarity between RyfA1 and RyfB1, four are contained within the RyfA1 variable region, providing a potential mechanism for specificity of regulation. However, complementary nucleic acids surrounding those included in the variable region are conserved between RyfA1 and RyfA2, a finding that might suggest that RyfB1 could downregulate both RyfA1 and RyfA2. This prediction is not supported by the experimental data presented above, which demonstrate that RyfB1 overproduction inhibits only RyfA1 levels (Figure 8). While the observed specificity may simply be due to the number of complementary nucleotides (RyfB1 has a greater number of complementary nucleotides to RyfA1 than RyfA2), the secondary structure of the RyfA molecules may also influence binding activity. Indeed, the variable region of RyfA1 is located almost entirely in a single-stranded region, indicating that if RyfA1 and RyfB2 interact directly, initial pairing between these two molecules may occur at the single-stranded variable region [19], thereby mediating the specificity of RyfB1 to modulate the levels of RyfA1 and not that of RyfA2. Further studies will be needed to confirm and understand this predicted interaction, as well as the specific molecular mechanism by which RyfA1 modulates the steady-state level of *ompC* in *S. dysenteriae*. While OmpC is necessary for cell-to-cell spread in *S. flexneri*, the protein also plays a role in the survival of gastrointestinal bacteria [30,34]. The relatively small porin size of OmpC (as compared to that of OmpF) protects the bacteria by slowing the diffusion of biosalts and toxins [35,36,37]. While *ompC* is regulated by numerous factors and regulatory networks [34], the additional control of *ompC* by RyfA1, whether it be through impacting timing of expression or the total amount of OmpC, may endow additional benefits to enteric pathogens containing this version of the RyfA molecule when faced with the harsh gut environment.

Many species of *Escherichia* and all species of *Shigella* contain a *ryfA* gene; however, few species contain both sibling copies, provoking interesting questions such as: what gave rise to either a single *ryfA1*-like iteration, a single *ryfA2* iteration or the sibling pair? Why is the presence of an *ryfA1*-like gene associated with enteropathogens while the presence of a *ryfA2* gene is associated with non-pathogenic or uropathogenic isolates? Based on the conservation of all identified *ryfA* genes, it is interesting to speculate that at some time RyfA played a role in a conserved process. In this model, it is proposed that the most evolutionarily ancient form of RyfA is that of RyfA2, the form of the regulator most highly represented in non-pathogenic enterobacteria, species from which pathogenic enterobacteria evolved. For pathogenic species, the harsh environment of the gut provides a powerful selection pressure, one not experienced by non-pathogens or uropathogens, and one that over time may have selected for a gene duplication event of *ryfA* [38]. For this scenario to be plausible, the gene duplication event must have provided a selective advantage to the organisms having experienced it. Based on the presented studies, it is possible that the presence of a second RyfA (RyfA1-like) molecule afforded the bacteria more precise control of OmpC production, an advantage that likely increased survival rates of the enteropathogens [39]. Based on the phylogenetic tree of *Shigella* and *E. coli* species, the gain of *ryfA1*-like genes is likely to have occurred concurrently, yet independently, in different species [40]. Loss of the original *ryfA* gene in some pathogenic species, an event leaving just the *ryfA1*-like gene, may have occurred later when acquired mutations afforded the RyfA1-like molecule the ability to regulate both the conserved process and *ompC*. Continued investigations will shed light on the evolutionary history of RyfA1 and RyfA2, as well as the relative functions of each.

This study is the first in what is likely to be a series of studies to fully elucidate the molecular mechanisms and physiological consequences of the complex regulatory network revealed here. While it is clear that RyfB1 influences RyfA1 levels and that RyfA1, in turn, influences *ompC* levels, many interesting questions remain. The exact molecular interplay between RyfA1/RyfB1 and *ompC* is the subject of current investigation. While at this point in time we cannot rule out the possibility that *ryfA1* encodes a small protein under the conditions we tested [31,32,41], our data thus far support the hypothesis that both RyfA1 and RyfB1 function as sRNA regulators. Specifically, the data presented are consistent with the model of a regulatory network in which RyfB1 functions to modulate the levels of RyfA1, which in turn functions to regulate *ompC* levels. One sRNA regulating another sRNA is not unprecedented; however, this is the first incidence of such regulation described in *Shigella* [42]. An additional level of regulation by sRNA/sRNA interactions could be advantageous to a pathogen that must survive fluctuating hostile conditions by allowing for rapid and receptive changes in transcript levels.

## Supplementary Materials

The following are available online at www.mdpi.com/2073-4425/8/2/50/s1, Figure S1: Sequence alignment comparing *ryfA* across isolates, Figure S2: Pairwise comparison of *ryfA* across *Shigella* and *Escherichia* isolates, Table S1: Primers and Probes Used, Table S2: Strains and Plasmids Used.
