# Supplementary material for: Sibling sRNA RyfA1 Influences Shigella dysenteriae Pathogenesis
Source: Genes (Basel). 2017 Jan 26;8(2):50. doi: 10.3390/genes8020050 (PMC5333039; doi:10.3390/genes8020050)
Supplement: Supplementary file 1 [file genes-08-00050-s001.pdf]

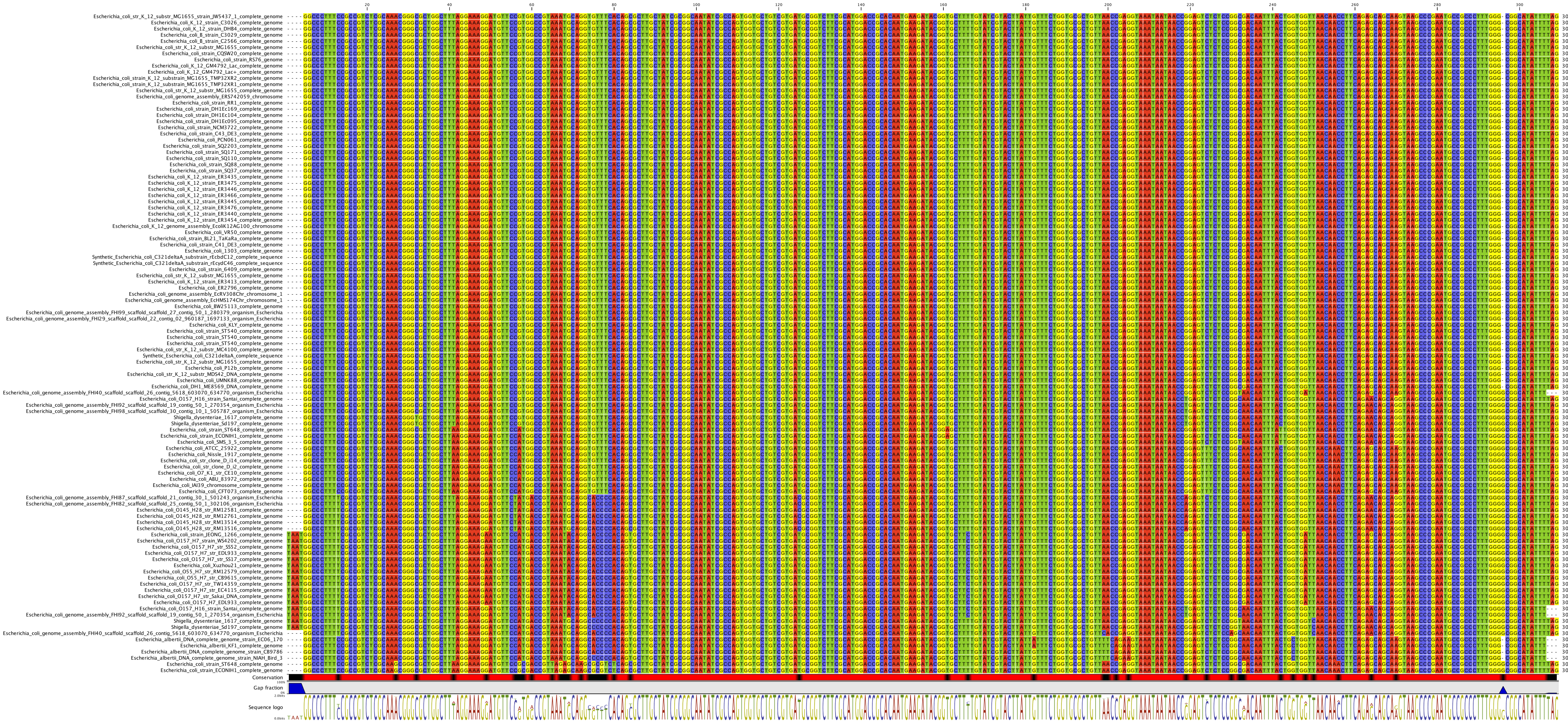

Figure S1: Sequence alignment comparing *ryfA* across isolates. All species carrying a *ryfA* gene from the NCBI or ShiBASE databases were compared for sequence similarity. Different colors indicate different nucleotides (yellow = guanine, red = adenine, blue = cysteine, green = thymine). The conservation black and red bar indicates where differences exist between species. The gap fraction (blue bar) shows where a gap occurs in the sequence from one isolate to another. Lastly the sequence logo shows nucleotide similarity relative to the height of the actual letter.

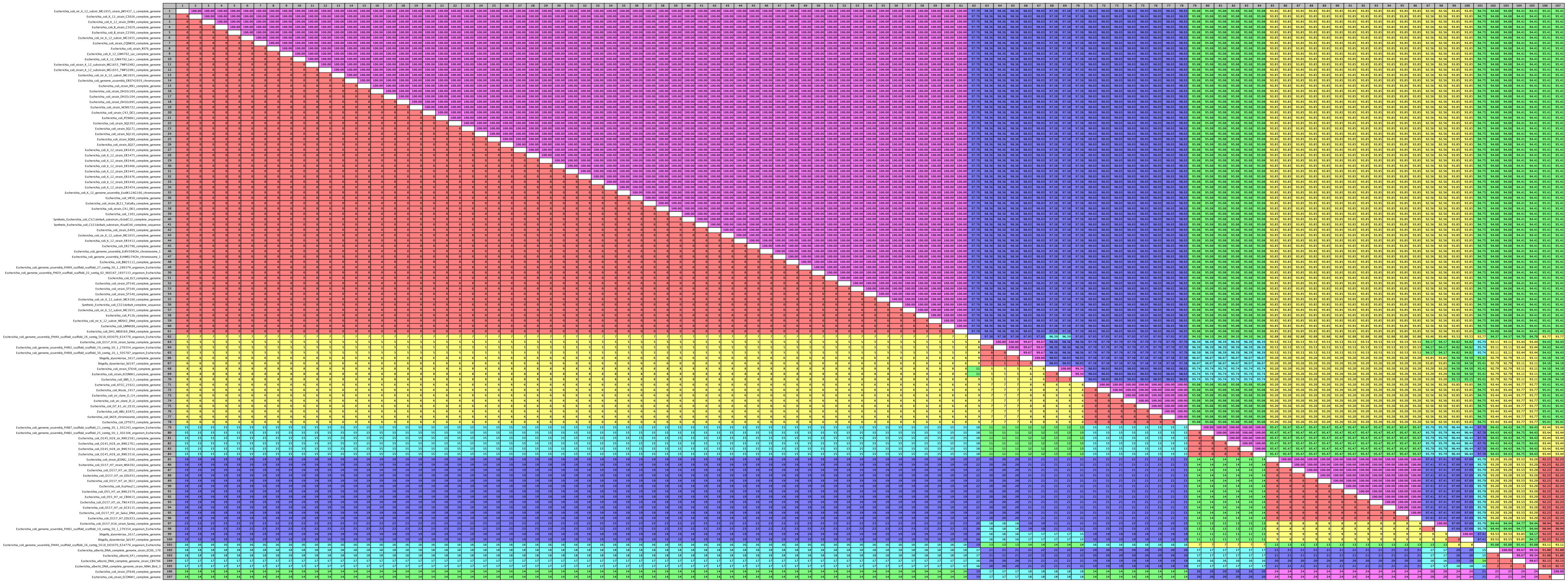

Figure S2: Pairwise comparison of *ryfA* across *Shigella* and *Escherichia* isolates. When each *ryfA* gene is compared to every other *ryfA* gene, no two genes are less than 90% identical, highlighting the importance of *ryfA* in *Shigella* and *Escherichia*. The darker hues indicate increased similarity (purple, blue, green) while the lighter hues indicate less sequence similarity (red, yellow). .
