# Supplementary material for: Sibling sRNA RyfA1 Influences Shigella dysenteriae Pathogenesis
Source: Genes (Basel). 2017 Jan 26;8(2):50. doi: 10.3390/genes8020050 (PMC5333039; doi:10.3390/genes8020050)
Supplement: Supplementary file 2 [file genes-08-00050-s002.docx]

Supplementary Materials: Sibling sRNA RyfA1 Influences *Shigella dysenteriae* Pathogenesis

Megan E. Fris, William H. Broach, Sarah E. Klim, Peter W. Coschigano, Ronan K. Carroll, Clayton C. Caswell and Erin R. Murphy

**Table S1.** Primers and probes used.

| **Name** | **Sequence** | **Use in this Study** |
| --- | --- | --- |
| *Primer* |  |  |
| RT ryfA1 for | CGTAAATCGAGGCCCCC | RT-PCR |
| RT ryfA2 for | GGCCGTAAATGCAGGTGTTT | RT-PCR |
| RT ryfA conserved rev | CTTACCTGCTGTTCTGAAGGTTG | RT-PCR |
| RyfA1-for | TAGCCAATTGCTTAATGGCCCTTTTCGC | Construction of pRyfA1 |
| RyfA1-rev | CCAAGCTTCCTGATTTTGGCGG | Construction of pRyfA1 |
| ryfA TM for | AAACGGGTGCTGGCTTTA | TaqMan primer qRT-PCR |
| ryfA TM rev | CAGCACCACTGGCGATATT | TaqMan primer qRT-PCR |
| rrsA TM for | GAGTTAGCCGGTGCTTCTT | TaqMan primer qRT-PCR |
| rrsA TM rev | GGCCTTCGGGTTGTAAAGTA | TaqMan primer qRT-PCR |
| RyfB1 for w_MfeI | TAGCCAATTGCACTGTGGGGTGCCTG | Construction of pRyfB1 |
| RyfB1 rev w_SacI | GAGCTCATGGAGTGAATGGGGCG | Construction of pRyfB1 |
| RyfB1 Left TM | GGGGTGCCTGCGTTGCTC | TaqMan primer qRT-PCR |
| RyfB1 Right TM | ATTTACCGGTTGAGCCATTG | TaqMan primer qRT-PCR |
| ompC-RT-f | TTT GCT GTT CAG TAC CAG GG | Sybrgreen primer qRT-PCR |
| ompC-RT-r | ATAATGGATAGATCCGCCAACG | Sybrgreen primer qRT-PCR |
| RyfA1-prom-1 | TCTTAATGGCCCTTTTCGCCGTCTCGCAAACGGGCGCTGGCTTTAGGAAAGGATGTTCCATGG | Construction of p5′UTR RyfA1 |
| RyfA1-prom-2 | CTAGCCATGGAACATCCTTTCCTAAAGCCAGCGCCCGTTTGCGAGACGGCGAAAAGGGCCATTAAGATGCA | Construction of p5′UTR RyfA1 |
| RT-RyfB1-L | GGGGTGCCTGCGTTGCTC | Sybrgreen primer qRT-PCR |
| RT-RyfB1-R | ATTTACCGGTTGAGCCATTG | Sybrgreen primer qRT-PCR |
| *Probe* |  |  |
| ryfA conserved probe | GCACCACTGGCGATATTGCCGCGATACGAAGC | Northern Blot |
| ryfA1 Fam probe | 6FAM-TAAATGCAGGCC/ZEN/CCCCACAGTGCTT-MGB-NFQ | TaqMan qRT-PCR Probe |
| ryfA2 Fam probe | 6FAM-TAAATGCAGGTG/ZEN/TTTCACAGCGCTT-MGB-NFQ | TaqMan qRT-PCR Probe |
| rrsA Fam probe | 6FAM-ACTCCCTTCC/ZEN/TCCCCGCTGAA-MGB-NFQ | TaqMan qRT-PCR Probe |
| RyfB1 probe | 6FAM-CACAAGTCA/ZEN/ACCTGCTGGAA-MGB-NFQ | TaqMan qRT-PCR Probe |

**Table S2.** Strains and plasmids used.

| **Strain or Plasmid** | **Description** | **Source** |
| --- | --- | --- |
| *Escherichia coli* strains |  |  |
| DH5α |  | Life Technologies |
| *Shigella dysenteriae s*trains |  |  |
| O-4576S1-G | Wild type *S. dysenteriae* | Murphy and Payne, 2007 [15] |
| Plasmids |  |  |
| pQE2 | Expression Vector | Qiagen |
| pRyfA1 | *ryfA1* expression vector | This study |
| pRyfB1 | *ryfB1* expression vector | This study |
| pXG-10 | Translational *gfp* reporter | Urban and Vogel, 2007 [2] |
| pXG-0 | Negative control for *gfp* reporter | Urban and Vogel, 2007 [2] |
| pXG-1 | Positive control for *gfp* reporter | Urban and Vogel, 2007 [2] |
| p5′UTR RyfA1 | putative *ryfA1* 5′ untranslated region | This study |
